# DARIER’S DISEASE WITH PERIFOLLICULAR HYPOPIGMENTATION

**DOI:** 10.4103/0019-5154.70685

**Published:** 2010

**Authors:** L Sornakumar, C R Srinivas

**Affiliations:** *From the Department of Dermatology, PSG Hospitals, Peelamedu, Coimbatore, India. E-mail: drsornakumar@yahoo.com*

**Figure 1:**
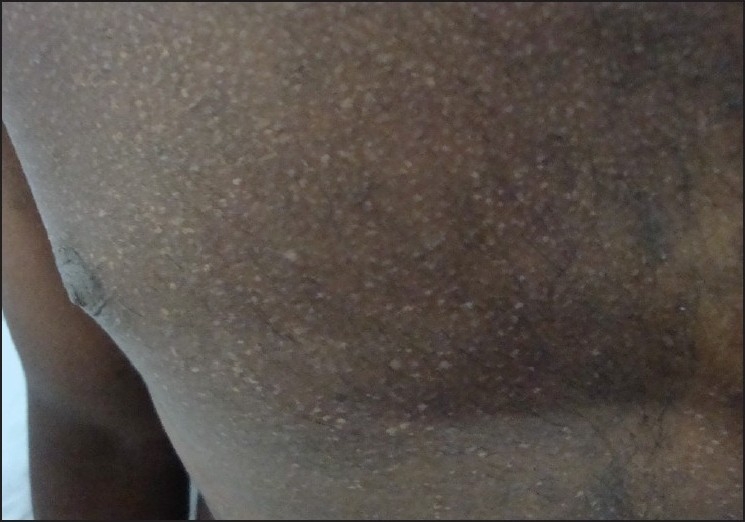
Perifollicular hypopigmented macules over the trunk

**Figure 2:**
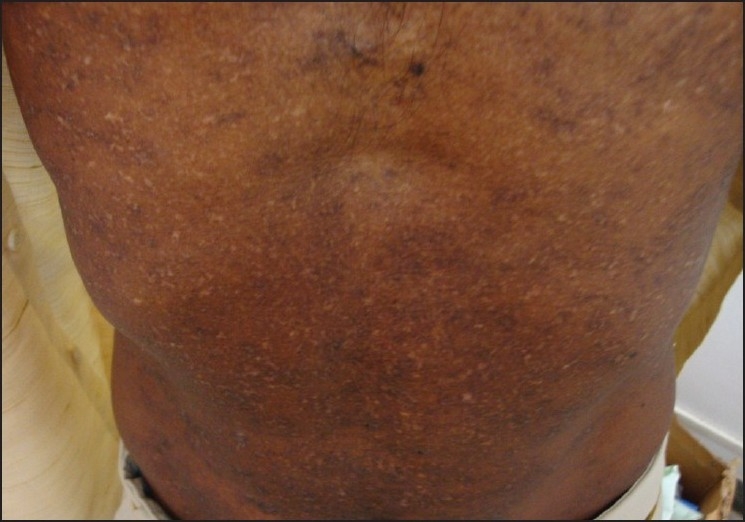
Similar perifollicular hypopigmented macules in patient’s father

Sir,

A 22-year-old male patient came with asymptomatic white lesions over forehead, chest, back and extremities of four years duration. Lesion started as asymptomatic hypopigmented macule over the chest [Figure 1] which spread to other areas over a period of four years. There was no history of any drug intake prior to the onset of the lesions. Before presenting to us, he was treated with one dose of oral antifungal and oral steroids daily for three months. He developed acneiform lesions over the chest and back. His father had similar hypopigmented lesions since child hood [Figure 2]. His mother and younger sister were not having any similar skin lesions. On examination, he showed multiple perifollicular 3-4 mm hypopigmented lesions over his face, trunk, back and over the extremites. Genital and oral mucosa were not involved. Hair, nails, palms and soles were not involved.

**Figure 3:**
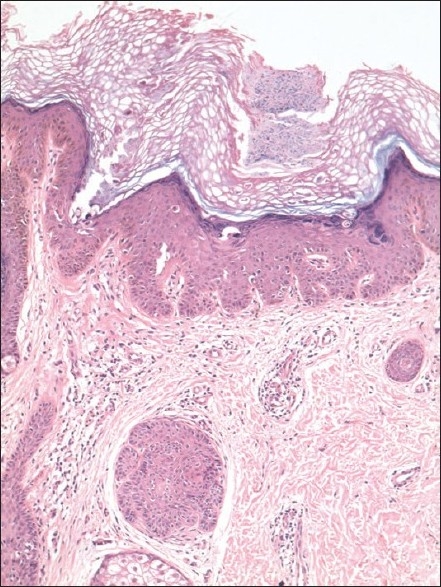
Biopsy revealing suprabasal acantholysis with corps ronds and grains (H and E stain, ×100)

Biopsy showed epidermal hyperkeratosis with cup-shaped invagination over sebaceous unit. The invagination contained parakeratotic layer with focal suprabasal acantholysis. Corps ronds and grains were seen suggesting a diagnosis of Darier’s disease [Figure 3].

Darier’s disease is a rare autosomal dorminant genodermatosis characterized by suprabasal acantholytic dyskeratosis. The distinctive lesions are skin colored, yellow brown or brown firm rough papules in the seborrhoeic areas such as scalp, face ad trunk. Other common skin manifestations are flexural papillomatous or vegetating lesions, acrokeratosis verruciformis over hands and feet, palmar and plantar pits. Oral mucosal findings include cobble-stone appearance of the buccal mucosa and leucoplakia. Characteristic nail changes include red or white longitudinal bands often ending in a pathogonomic notch at the free margin of the nail. Other rare variants are linear, unilateral,[1] bullous and hypopigmented variants. Biopsy will have dyskeratotic cells(corp ronds, grains) and suprabasal acantholysis.

Small leukodermic macules were first described by Goddal and Richmond in 1965. Hypopigmented macules in Darier’s disease is rare.[2] Hypopigmented perifollicular macules may be misdiagnosed as tinea versicolor, lichen simplex et atropicus, idiopathic guttate hypomelanosis or follicular vitiligo. According to Tolat *et al*.[3] familial hypopigmented Darier’s differ from usual Darier by involvement of gluteal region and back. Nails, palms and mucosa were unaffected in familial type. Similar findings were found in our case. Electron microscopy has demonstrated an absence of heavily melanized melanosomes (Stage III, IV) from the guttate leukodermic lesions.[4] A faulty keratinization interfering with melanosome transfer and an overall disruption of the “epidermal melanin unit” may contribute to this strange focal, macular depigmentation of Darier’s disease. We report this case because of clinical rarity.
